# Core auditory processing deficits in primary progressive aphasia

**DOI:** 10.1093/brain/aww067

**Published:** 2016-04-09

**Authors:** Manon Grube, Rose Bruffaerts, Jolien Schaeverbeke, Veerle Neyens, An-Sofie De Weer, Alexandra Seghers, Bruno Bergmans, Eva Dries, Timothy D. Griffiths, Rik Vandenberghe

**Affiliations:** ^1^ 1 Institute of Neuroscience, Medical School, Newcastle University, Newcastle-upon-Tyne, UK; ^2^ 2 Machine Learning Group, Department of Computer Science, Berlin Institute of Technology, Berlin, Germany; ^3^ 3 Laboratory for Cognitive Neurology, KU Leuven Department of Neurosciences, Belgium; ^4^ 4 Neurology Department, University Hospitals Leuven, Leuven, Belgium; 5 5 Neurology Department, AZ Sint-Jan Brugge-Oostende AV, Brugge, Belgium; ^6^ 6 Wellcome Centre for Neuroimaging, University College London, UK; ^7^ 7 Alzheimer Research Centre KU Leuven, Leuven research Institute for Neuroscience and Disease, University of Leuven, Belgium

**Keywords:** semantic dementia, progressive non-fluent aphasia, pitch, rhythm, timbre

## Abstract

The extent to which non-linguistic auditory processing deficits may contribute to the phenomenology of primary progressive aphasia is not established. Using non-linguistic stimuli devoid of meaning we assessed three key domains of auditory processing (pitch, timing and timbre) in a consecutive series of 18 patients with primary progressive aphasia (eight with semantic variant, six with non-fluent/agrammatic variant, and four with logopenic variant), as well as 28 age-matched healthy controls. We further examined whether performance on the psychoacoustic tasks in the three domains related to the patients’ speech and language and neuropsychological profile. At the group level, patients were significantly impaired in the three domains. Patients had the most marked deficits within the rhythm domain for the processing of short sequences of up to seven tones. Patients with the non-fluent variant showed the most pronounced deficits at the group and the individual level. A subset of patients with the semantic variant were also impaired, though less severely. The patients with the logopenic variant did not show any significant impairments. Significant deficits in the non-fluent and the semantic variant remained after partialling out effects of executive dysfunction. Performance on a subset of the psychoacoustic tests correlated with conventional verbal repetition tests. In sum, a core central auditory impairment exists in primary progressive aphasia for non-linguistic stimuli. While the non-fluent variant is clinically characterized by a motor speech deficit (output problem), perceptual processing of tone sequences is clearly deficient. This may indicate the co-occurrence in the non-fluent variant of a deficit in working memory for auditory objects. Parsimoniously we propose that auditory timing pathways are altered, which are used in common for processing acoustic sequence structure in both speech output and acoustic input.

## Teaser

The extent to which deficits in non-verbal auditory processing contribute to the clinical phenotype of primary progressive aphasia (PPA) is unclear. Grube *et al.* reveal impairments in processing the timing of brief sequences of non-linguistic stimuli, particularly in the non-fluent variant, indicative of a core central auditory impairment in PPA.

## Graphical abstract

**Figure:**
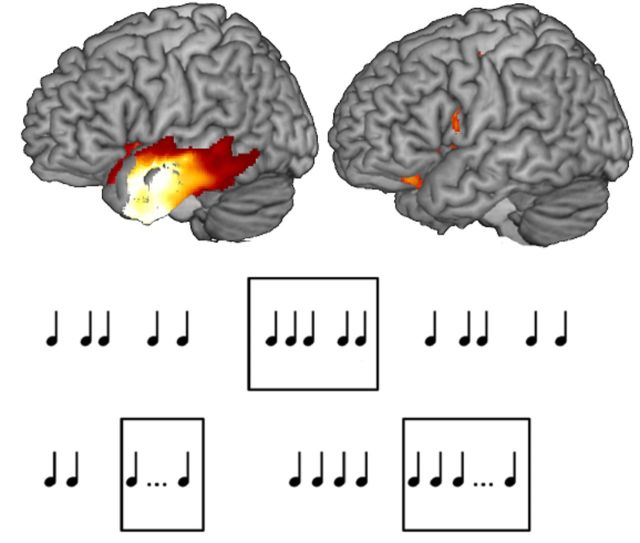
The extent to which deficits in non-verbal auditory processing contribute to the clinical phenotype of primary progressive aphasia (PPA) is unclear. Grube *et al.* reveal impairments in processing the timing of brief sequences of non-linguistic stimuli, particularly in the non-fluent variant, indicative of a core central auditory impairment in PPA.

## Introduction

Primary progressive aphasia (PPA) is characterized by progressive word finding difficulties caused by selective neurodegeneration of the language network (Mesulam *et al.*, 2014). Depending on the subtype (Gorno-Tempini *et al.*, 2011), the word finding problems may be accompanied by word comprehension and object recognition deficits, as in the semantic variant (SV) (Hodges *et al.*, 1992); speech apraxia or agrammatism may be most prominent, as in the non-fluent/agrammatic variant (NFV) (Grossman, 2012); or phonological working memory deficits may cause deficits repeating series of numbers and complex sentences, as in the logopenic variant (LV) (Gorno-Tempini *et al.*, 2004, 2008; Mesulam *et al.*, 2012).

While the core clinical phenotype in PPA is by definition dominated by the verbal communication difficulties, the concomitance of non-verbal deficits of object recognition and object knowledge are a well-documented and defining feature of the semantic variant (Hodges *et al.*, 1992; Gorno-Tempini *et al.*, 2011). In subtypes distinct from the semantic variant, experimental studies have unveiled deficits in non-verbal domains e.g. related to ideomotor praxis (Adeli *et al.*, 2013) and gesture discrimination (Nelissen *et al.*, 2010). The study of combined verbal and non-verbal deficits may yield a unique view on ontogenetic and neuroanatomical bonds between language and specific non-verbal processes in the intact brain and inform hypotheses about the pathogenesis of the language dysfunction.

The purpose of the current study was 2-fold: at the basic neuroscience level to evaluate how language and speech relate to core auditory analysis of pitch, rhythm and timbre; and at the clinical level, to examine how deficits in non-verbal sound processing relate to the pathogenesis of clinical symptoms in PPA. For pitch (Patterson *et al.*, 2002), time (Teki *et al.*, 2011) and timbre (Overath *et al.*, 2008, 2010), extensive processing occurs in areas beyond primary auditory cortex, especially at higher levels of complexity or longer temporal windows of integration.

Compared to earlier studies (Bozeat *et al.*, 2000; Warren *et al.*, 2005; Goll *et al.*, 2010, 2011; Hailstone *et al.*, 2011; Hsieh *et al.*, 2011, 2012; Omar *et al.*, 2011; Rohrer *et al.*, 2012; Fletcher *et al.*, 2013), our approach was unique in the systematic nature of the investigation of the three domains within the same patients across the range of PPA subtypes. Moreover, we focused entirely on auditory processing levels of non-linguistic stimuli preceding the association with meaning (be it verbal meaning, emotional content or identity). As a third important distinction from previous work, we assessed pitch and time judgements for sequences of tones up to seven tones while previous paradigms when using non-linguistic stimuli focused on single tones or pairs of tones.

The tasks and stimuli we used have been previously applied in children and young adults with typical and atyical language development (Foxton *et al.*, 2003, Grube *et al.*, 2013, 2014), patients with congenital amusia (Foxton *et al.*, 2004), patients with Wernicke’s aphasia (Robson *et al.*, 2013) and patients with cerebellar degeneration or basal ganglia dysfunction (Grube *et al.*, 2010; Cope *et al.*, 2014).

The non-linguistic acoustic stimuli used were designed to have structure that is relevant to speech and language processing at different levels from individual phonemes to sentences. In the pitch and time domain the structure varied between single pitch or time intervals and sequences of pitch or time intervals. In the timbre domain we also manipulated features over different timescales that correspond with phoneme or sentence-level analysis. In both domains of pitch and time/rhythm, sequence processing has been related to prosodic processing and segmentation processes in speech and to language skills in children (Grube *et al.*, 2014) and young adults (Foxton *et al.*, 2003; Grube *et al.*, 2014). In the timbre domain, frequency modulation (FM) and more complex spectro-temporal modulation patterns (dynamic modulation, DM) are relevant to speech perception (Drullman, 1995; Shannon *et al.*, 1995; Boemio *et al.*, 2005; Giraud and Poeppel, 2012). Specifically, 2 Hz FM corresponds to a temporal window relevant to slow, prosodic variations and 40 Hz FM to fast phonemic variations. The dynamic modulation tasks assess mechanisms relevant to the processing of complex spectral patterns that change over time, like formant changes in speech perception (Chi *et al.*, 1999*a*; Elliott and Theunissen, 2009). Dynamic modulation detection has been previously shown to relate to speech comprehension in acquired Wernicke’s aphasia (Robson *et al.*, 2013) and dynamic modulation discrimination scores correlate with speech and language skills in children with dyslexic traits (Grube *et al.*, 2013).

We assessed how auditory perception across the domains of pitch, time and timbre relates to specific aspects of speech and language skill in PPA.

## Materials and methods

### Subjects

The study was approved by the Ethics Committee, University Hospitals Leuven. All participants provided written informed consent in accordance with the Declaration of Helsinki.

PPA patients were recruited via the memory clinic University Hospitals Leuven (Table 1). A consecutive series of 23 patients who fulfilled the international consensus criteria for PPA (Gorno-Tempini *et al.*, 2011) enrolled for the experiment (Table 1). Five of the subjects had to be excluded for the following reasons: hearing loss (*n* = 1); lack of ability to perform the experimental tasks according to practice runs due to disease severity (*n* = 2); lack of cooperation (*n* = 1); unique phenotype (foreign accent syndrome). The remaining 18 participants were able and sufficiently cooperative to undergo the extensive psychoacoustic testing and produce reliable data.

**Table 1:** Demographics and neuropsychological assessment

Case	1	5	7	10	12	14	17	19	6	13	15	20	21	22	16	4	9	11	
Age	76	70	61	64	64	48	58	69	52	79	71	78	72	63	71	57	64	62	
Sex	M	F	M	F	M	F	F	F	F	F	M	M	F	F	M	F	M	F	
Education level (years)	14	12	17	12	8	12	10	7	17	8	15	17	12	15	15	15	17	15	
PPA subtype	SV	SV	SV	SV	SV	SV	SV	SV	NFV	NFV	NFV	NFV	NFV	NFV	LV	LV	LV	LV	
Disease duration (years)	6	1,5	3	3	3	6	2	5	2	5	1,5	2,5	2,5	5	1	0,5	3	1,5	
FDG PET	+	+	+	+	+	+	+	+	-	+	+	**+**	-	+	-	+	+	+	
PIB PET SUVR_comp_	1,15	-	-	-	-	-	-	-	1,2	-	-	**-**	-	1.16	-	**1,81**	**2,24**	-	
CSF Aβ42 (pg/ml)	-	1330	-	-	-	-	-	733	-	1028	-	816	1060	865	-	664	-	**516**	
CSF total tau (pg/ml)	-	269	-	-	-	-	-	262	-	312	-	195	**424**	183	-	183	-	**>1200**	
BNT (/60)	**34**	**22**	**7**	**10**	**20**	**12**	**35**	**16**	58	48	55	48	**30**	**41**	53	56	**41**	53	
**PALPA comprehension**																			
Auditory WP matching (/40)	38	**36**	**31**	**29**	**31**	**27**	40	**30**	40	40	**37**	39	**36**	**38**	40	40	40	40	
Verbal assoc.-sem. (/30)	27	**17**	**18**	**22**	**14**	**24**	27	**21**	28	**22**	28	**23**	**19**	**24**	28	28	**23**	27	
**AAT comprehension**																			
Auditory word (/30)	**19**	**15**	**11**	**19**	**19**	**11**	**21**	**14**	30	27	27	24	**20**	**21**	28	30	29	25	
Auditory sentence (/30)	26	**20**	25	**19**	**20**	25	**21**	**24**	30	**24**	25	**12**	**23**	25	30	30	27	25	
Written word (/30)	**20**	**18**	**13**	**11**	**13**	**19**	**24**	**14**	30	30	**25**	**23**	**21**	28	30	30	30	**26**	
Written sentence (/30)	25	**21**	24	**23**	**22**	27	27	30	30	24	25	**18**	**21**	30	30	30	28	30	
**PALPA repetition**																			
Single word (/80)	**72**	**78**	**78**	**79**	**71**	**79**	**76**	**78**	**76**	**74**	**74**	**74**	**69**	80	80	80	**77**	80	
Pseudoword (/80)	**55**	**71**	**74**	**65**	**56**	80	**72**	77	**55**	**59**	**40**	**54**	**30**	79	**63**	**74**	**69**	79	
**AAT repetition**																			
Total score (/150)	147	147	141	150	143	150	148	146	134	**125**	**123**	144	**102**	148	140	147	145	150	
Phonemes (/30)	30	28	29	30	28	30	30	30	28	29	30	29	26	30	27	30	28	30	
Single words (/30)	27	29	29	30	27	30	30	28	**26**	**24**	**21**	30	28	30	30	30	29	30	
Cognate words (/30)	30	30	30	30	30	30	30	29	28	**22**	29	28	**24**	30	29	30	30	30	
**Object decision**																			
Easy B (/32)	**23**	**18**	**20**	**21**	26	26	31	**23**	31	30	31	26	**18**	**22**	31	29	31	31	
Hard A (/32)	**20**	**20**	**18**	**18**	**18**	**17**	27	**17**	26	25	26	29	**21**	28	26	25	25	**24**	
PPT Pictures (/52)	**37**	**39**	**40**	**35**	**40**	**46**	50	**43**	52	51	50	47	46	48	50	51	51	52	
Digit Span forward	6	**3**	-	6	6	6	5	5	6	**3**	**4**	6	**2**	**4**	-	**4**	7	5	
CPM (/36)	29	**24**	36	31	29	35	36	34	31	**24**	**24**	30	**12**	32	33	35	33	31	

FDG PET = ^18^F-fluorodeoxyglucose positron emission tomography; PIB PET SUVR_comp _= Pittsburgh compound-B PET standardized uptake value ratio in the composite cortical volume of interest; WP = word-picture; PPT = Pyramids and Palm Trees test.

– = Test was not performed.

Bold indicates >2 SD below the mean according to published norms.

Before the study, each PPA patient was classified according to the Gorno-Tempini *et al.* (2011) recommendations. The classification relied on the clinical evaluation by an experienced neurologist (R.V.), in combination with neurolinguistic assessment and clinical MRI, as well as, where available, ^18^F-fluorodeoxyglucose PET (^18^F-FDG PET), CSF Alzheimer’s disease biomarkers and ^11^C-Pittsburgh compound B amyloid PET (Table 1). Eight cases were classified as SV, six as NFV, and four as LV (Table 1) (Gorno-Tempini *et al.*, 2011). The SV and LV groups each showed a relatively homogeneous classical phenotype (Gorno-Tempini *et al.*, 2011). All NFV cases had speech apraxia with buccolingual apraxia. In one NFV case (Case 6), speech apraxia was by far the most prominent clinical abnormality, with no apparent agrammatism and preserved comprehension. According to the Josephs *et al.* (2013) terminology, this case could also possibly be classified as primary progressive apraxia of speech, at least during the initial disease phase when she underwent the experimental tests. NFV Cases 13 and 15 had agrammatism in addition to speech apraxia. Cases 20–22 also had word comprehension deficits on detailed testing besides agrammatism and speech apraxia with, in addition, object recognition problems in Cases 21 and 22 (Table 1). While we included these subjects in the NFV group, the latter three cases would also fit into the recently defined ‘mixed’ subtype (Mesulam *et al.*, 2012, 2014).

An ^18^F-FDG PET was available in 14 of the cases and a volumetric MRI in 17 cases (Case 10 had claustrophobia and received ^18^F-FDG PET only). The NFV group showed volume loss and hypometabolism in left frontal opercular, left anterior insular and left supplementary motor cortex (Fig. 2A). The SV cases showed anterior temporal and orbitofrontal abnormalities, strictly conforming to the expected pattern (Gorno-Tempini *et al.*, 2011; Rogalski et al., 2011) (Fig. 2B). The LV group showed a more heterogeneous pattern, with clear posterior temporal hypometabolism in Case 9, premotor involvement in Case 11 and ventral temporal hypometabolism in Case 4.

Twenty-eight age- and education-matched cognitively intact control subjects [half male, age range 51–76 years, mean 62.2, standard deviation (SD) 7.8; education range 9–22 years, mean 14.0, SD 2.8] completed the same experimental psychoacoustic test battery as the patients. All subjects also underwent a full neuropsychological assessment. For the purpose of comparing brain volume in the PPA subtypes with that of a normal control group, we made use of a large group of 86 healthy age- and gender-matched control subjects (47 male; age range 53–76, mean 64.9, SD 5.9 years). This normative MRI cohort included, among others, 25 of the controls from the psychoacoustic experiment (the remaining three controls refused MRI or had a medical contraindication).

Hearing sensitivity was measured in all participants using a clinical Bekesy-type audiometer for frequencies of 0.5, 1, 2, 4, and 8 kHz, on the left and right ear, respectively. In the pure-tone audiograms, all subjects were able to detect stimuli of up to 1000 Hz below a hearing level of 30 dB on at least one side (Supplementary Table 1). There were no significant differences between left and right ear hearing levels (two-tailed *t-*test, *P* = 0.14).

### Neuropsychological protocol

Confrontation naming was assessed by means of the Boston Naming Test (BNT) (Kaplan *et al.*, 1983; Marien *et al.*, 1998). We assessed single word comprehension and repetition using tests selected from the Psycholinguistic Assessment of Language Processing in Aphasia (PALPA) (Kay *et al.*, 1992; Bastiaanse *et al.*, 1995) and the Akense Afasie test (Graets *et al.*, 1992) (Table 1). Non-verbal executive functioning was evaluated by means of the Raven’s Coloured Progressive Matrices. The neuropsychological tests used are provided in Table 1 and described in further detail in the Supplementary material. Although it was not part of the neuropsychological protocol of the study, we retrieved the digit span forward scores from the clinical patient files, where available (Table 1).

### Experimental tests of pitch, rhythm and timbre

**Figure 1:**
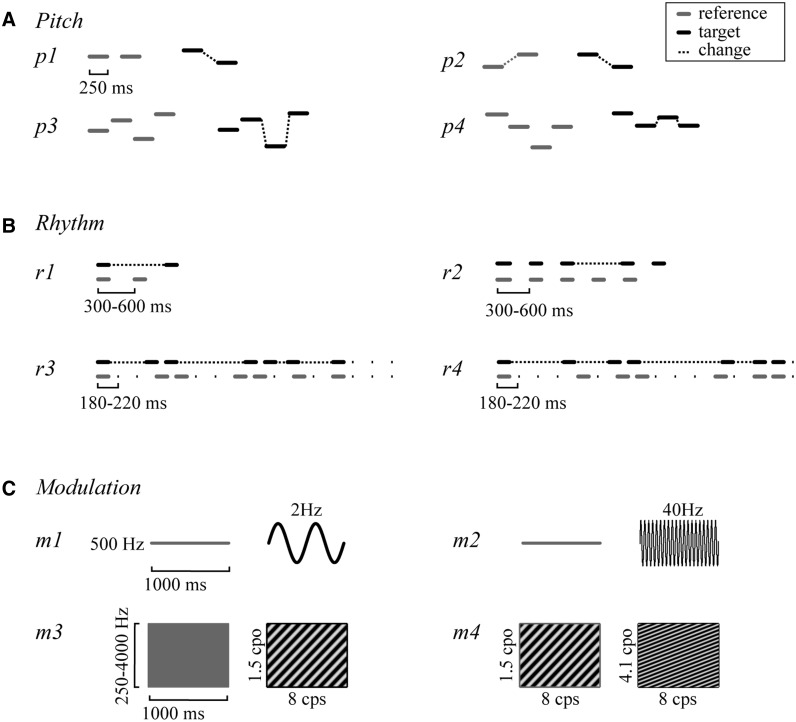
**Schematic illustration of experimental tasks, with one reference and one target example depicted per task.** (**A**) Pitch: basic change detection (p1); change-direction discrimination (p2); detection of a local change in pitch sequence (p3); detection of a global change in pitch sequence (p4). (**B**) Rhythm and timing: single time-interval discrimination (r1); isochrony deviation detection (r2); metrical pattern discrimination for a strongly (r3) and a weakly metrical sequence (r4). (**C**) Modulation (timbre): 2 Hz frequency modulation (FM) detection (m1); 40 Hz FM detection (m2); dynamic modulation (DM) detection (m3); dynamic modulation discrimination (m4). Note: *x-* and *y*-axes correspond to time and frequency throughout, but scales vary and are in part arbitrary.

The psychoacoustic battery consisted of four pre-existing tasks of pitch, rhythm and timbre each (Grube *et al.*, 2012, 2014). Ten of the 12 tasks used a two-alternative forced-choice adaptive paradigm following a two-down, one-up algorithm (Levitt, 1971). Two tasks (see below: p3, p4) used a same-different paradigm with fixed difficulty levels. A schematic representation of all tasks is provided in Fig. 1.

**Figure 2:**
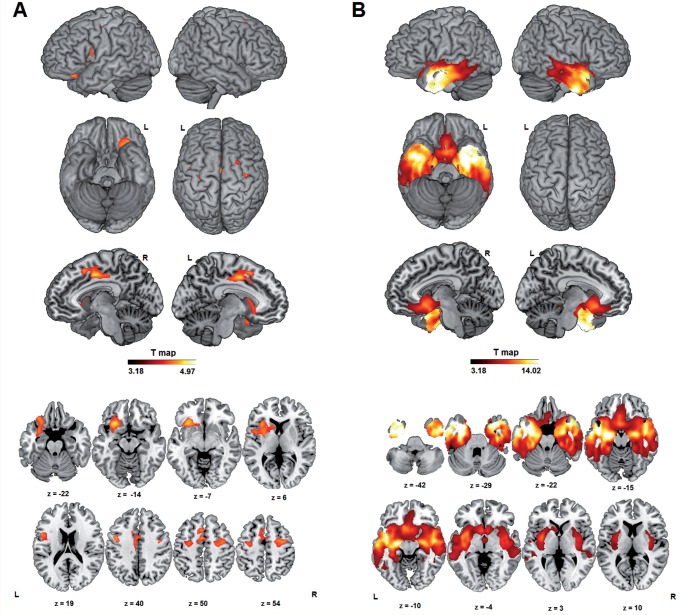
**Volumetric comparison between PPA subtypes and controls by means of one-way ANOVA with grey matter volume as dependent variable and four subgroups (NFV, SV, LV and controls) as between-subjects factor, corrected for age.** (**A**) NFV group versus controls. (**B**) SV group versus controls. T-maps projected on a rendered brain template with voxel-level uncorrected *P* < 0.001 and cluster-level FWE-corrected *P* < 0.05. We did not obtain any significant differences between our LV sample and controls at the pre-set threshold.

Depending on preference and ability, subjects responded verbally, by pressing keys on the keyboard, or by pointing to the corresponding interval on a graphical scheme reflecting the trial structure in front of them (a piece of paper in front of the subjects showing two or three circles numbered 1, 2 and 3). Each subject responded using a same response modality for all tasks. Prior to each task, we explained to the subject which domain (pitch, etc) was being probed. The instructions were repeated to the subject as needed until he or she confirmed they fully understood the task. We presented the instructions verbally, and also in a graphical way (Supplementary Fig. 1) and by simple examples. Next, subjects received a number of practice trials. The difficulty level of the practice trials was the same for all subjects at the start for each task. If an incorrect response was given on a practice trial, the subject received feedback on the nature of the error. Practice trials and instructions were repeated until five consecutive correct responses were recorded. If needed, the starting level was decreased in difficulty individually in patients. The difference between target and reference stimuli at the start of the test trials was the same as in the practice trials. If the subject indicated during the test trials that they had forgotten the instructions, the task was aborted, the instructions repeated, practice trials run again and the test phase then re-started.

Below follows a brief description of the psychoacoustic test battery, with more details provided in the Supplementary material for each of the domains.

#### Pitch

The basic ‘Change in pitch detection’ task (p1; 50 trials; AB) (Fig. 1A) required pure tones to be discriminated based on their frequency. On each trial, the subject was presented with two pairs of tones (AB) with a tone duration of 250 ms and 2000 ms interval between pairs. The target pair (A or B) contained a change in frequency (up or down). The task of the subject was to identify the pair that included the change in frequency. The outcome measure was the threshold obtained by adaptively adjusting the size of the change in frequency.

The ‘Pitch change direction discrimination’ task (p2; 50 trials; AB) (Fig. 1A) required two pairs of pure tones to be judged as ‘same’ or ‘different’ based on the direction of the changes in frequency within the pairs (same-different judgement). The frequency within either pair could either go ‘up’ or ‘down’, and the two pairs could thus either have the ‘same’ or ‘different’ directions. The outcome measure was the threshold obtained by adaptively adjusting the size of the change in frequency.

The ‘Local and global change in pitch detection’ tasks (p3, p4; 40 trials; AB) (Fig. 1A) used pitch sequences of four tones each of duration 250 ms, separated by an interstimulus interval of 2000 ms (in-between sequences) and required the subject to indicate whether two sequences were ‘the same’ or ‘different’ (adapted from Foxton *et al.*, 2003) (same-different judgement). In the ‘Local change detection’ task (p3), in the ‘different’ pairs, there was one change in frequency in the third or fourth note in the second sequence compared to the first, but the patterns of ‘ups and downs’ remained preserved. In the ‘Global change detection’ task (p4) in contrast, the change in frequency in the third or fourth tone caused a change also in the global contour, i.e. in the pattern of ‘ups and downs’. Each task contained 40 trials, including 20 ‘same’ and 20 ‘different’, with each reference sequence occurring once in a ‘same’ and once in a ‘different’ trial. The outcome measure was the score correct.

#### Rhythm and timing

All four rhythm and timing tasks (Fig. 1B) used 500 Hz, 100 ms pure tones. The outcome measure for each of the tasks was the threshold obtained by adaptively adjusting the difference between reference and target stimuli. The difference was measured relative (in per cent) to the duration or tempo of the reference.

The basic ‘Single time-interval duration discrimination’ task (r1; 50 trials; AB) (Grube *et al.*, 2010) required subjects to indicate which of two tone pairs comprised the ‘longer gap’ than the reference of varying duration (interonset interval of 300–600 ms).

In the ‘Isochrony deviation detection’ task (r2; 50 trials; AB) (Grube *et al.*, 2012), subjects were required to indicate which of two otherwise isochronous five-tone sequences contained a lengthening or ‘extra gap’. The reference sequence had an isochronous interonset interval ranging from 300 to 600 ms, the target had one lengthened interonset interval between the third and fourth tone. In the ‘Metrical pattern discrimination’ tasks (r3, r4; 50 trials; XAB) (Grube and Griffiths, 2009), subjects were required to decide which of three rhythmic sequences of seven tones each sounded ‘different’, or ‘wrong’, based on a distortion (or change) in the rhythm. The reference sequence had a strongly (r3) or a weakly (r4) metrical beat of 4.

### Modulation

The four tasks of timbre (modulation) perception (Fig. 1C; Grube *et al.*, 2012) included two FM detection tasks, one ‘spectro-temporal, dynamic modulation detection’ and one ‘dynamic modulation discrimination’ task, testing the processing of modulations thought to be relevant to speech (Witton *et al.*, 1998; Chi *et al.*, 1999; Talcott *et al.*, 2000; Schönwiesner and Zatorre, 2009; Grube *et al.*, 2012; Robson *et al.*, 2013). The outcome measure for each of these tasks was the threshold obtained by adaptively adjusting the amount or difference in modulation.

In the two FM detection tasks (m1, m2; 50 trials; AB) (Grube *et al.*, 2012), subjects were required to identify a tone modulated at a rate of 2 Hz (sounding ‘ringing or wobbly’) and 40 Hz (sounding ‘rough’), respectively, against a ‘flat-sounding’ unmodulated 500 Hz reference. On each trial, two 1000 ms tones were presented and subjects had to identify the modulated one. The dynamic modulation detection task (m3; 50 trials; AB) (Grube *et al.*, 2012) required the discrimination of a modulated (‘alien or laser-like’) target sound against an unmodulated reference. On each trial, two 1000 ms sounds were presented and subjects had to identify the modulated one. The dynamic modulation discrimination task (m4; 50 trials; AXB) (Grube *et al.*, 2012) required the discrimination of such spectro-temporal modulation sounds based on a difference in spectral modulation rate density.

### Volumetric MRI

All patients, except Case 10 (claustrophobia), and 86 healthy controls received a high resolution T_1_-weighted structural MRI on a 3 T Philips Intera system equipped with an 8-channel receive-only head coil (Philips SENSitivity Encoding head coil), using a 3D turbo field echo sequence. Further details are provided in the Supplementary material.

### Analysis procedures

#### Behavioural data

All psychoacoustic group data were tested for normality with the Lilliefors version of the Kolmogorov-Smirnoff test for composite normality. In cases where the outcome measure for a given task in its original format deviated from normality in controls or in patients, we log-transformed the data for both groups and used these as outcome measures to allow for parametric analyses at the group level.

At the group level, the psychoacoustic data were compared between the PPA group and the healthy controls using a one-sided independent-samples *t*-test with the threshold for significance set at *P *< 0.05, Bonferroni-corrected for multiple comparisons by the number of tests performed (*n* = 12). We compared the psychoacoustic test scores between the subtypes (SV, NFV and LV) using Kruskal-Wallis ANOVA followed by *post hoc* Tukey-Kramer pairwise comparison of subtypes (*P < *0.05).

At the individual level, each PPA patient’s performance was analysed in comparison to the group by using a modified *t*-test (Crawford and Garthwaite, 2007). For the comparison between each individual patient and the controls, to facilitate comparison between tasks and to enable Bonferroni correction, the exact *P*-values (estimated percentiles) calculated according to Crawford and Garthwaite (2007) were transformed into normalized *Z*-scores using the standard normal cumulative distribution function. The significance threshold was set to *Z* = 2.64 equalling a one-tailed significance level of *P *< 0.05, Bonferroni-corrected for the number of tests (*n* = 12).

The individual level of impairment was also verified with adjustment for potential contributing factors such as age or general cognitive capacity (as measured by Coloured Progressive Matrices) following the procedure developed by Crawford and Garthwaite (2006). As a first step, we performed a multiple linear regression analysis in the healthy controls for each of the psychoacoustic tests, with as outcome variable the psychoacoustic test score and as independent variables age and executive function. Variables that had a significant effect on any of the test scores in these multiple regression analyses in normal controls were used as covariates for which we adjusted when comparing the individual’s patient scores with the normal control group following the procedure developed by Crawford and Garthwaite (2006). Note that if a covariate had a significant effect on any of the 12 tests, it was included as a covariate in the regression equation for each of the 12 tests when comparing individual patients with controls.

Furthermore, we examined how accurately a machine learning-based classifier could assign the individual cases to one of the three clinical subtype groups (three classes: NFV, SV and LV) based on the set of 12 psychoacoustic test scores exclusively, and which of the psychoacoustic tests were most discriminative in this respect. We used a linear support vector machine (SVM) approach (C = Inf, alpha = 0) as implemented in Spider (version 1.71, http://people.kyb.tuebingen.mpg.de/spider/, Weston J., *et al.*, Max Planck Institute for Biological Cybernetics, Tübingen, Germany, running under Matlab version 2011b) for pairwise classification into every possible pair of subtypes (NFV-LV, NFV-SV, LV-SV). The details of this analysis procedure are described in the Supplementary material.

We further tested for a relationship between the psychoacoustic test scores and neuropsychological measures. Given the relatively large number of the conventional neuropsychological tests administered and the potential correlations between scores on these tests, we first conducted a factor analysis (SPSS Statistics 22, IBM) on the full neuropsychological dataset to reduce the number of correlations to be performed with the psychoacoustic scores. The dataset included the conventional neuropsychological test scores (Table 1) from all PPA patients plus the 28 age-matched controls who had undergone the same neuropsychological battery. The factor analysis procedure was identical to that used in previous studies (Vandenbulcke *et al.*, 2005; Molenberghs *et al.*, 2009; Nelissen *et al.*, 2010). Details of the factor analysis procedure are described in the Supplementary material. The individual factor scores of the PPA cases were correlated with their psychoacoustics scores using the Pearson correlation method throughout. The significance threshold was set at one-tailed *P *< 0.05, Bonferroni-corrected for multiple comparison by the number of tests (*n* = 12 times the number of factors).

Finally, we performed within the PPA group a Pearson regression analysis for each of the psychoacoustic test scores with symptom duration as the independent variable, and a similar analysis with audiometric hearing levels as the independent variable. We also performed a Spearman regression analysis for each of the psychoacoustic test scores with digit span forward as independent variable (from the patients in whom it was available; Table 1). The significance threshold was set at *P* < 0.05 corrected for the number of psychoacoustic tests (*n* = 12).

### MRI analysis

All procedures were carried out with Statistical Parametric Mapping 8 (SPM8, Wellcome Trust Centre for Neuroimaging, London, UK, http://www.fil.ion.ucl.ac.uk/spm) and the Voxel-Based Morphometry 8 toolbox (VBM8, http://dbm.neuro.uni-jena.de/vbm). High-resolution T_1_-weighted images were registered to the Montreal Neurological Institute (MNI) space and segmented into grey matter, white matter, and CSF (Ashburner and Friston, 2005). The normalized grey matter partitions were weighted (‘modulated’) to account for non-linear volume changes resulting from the normalization process. **‘**Non-linear modulation only’ was chosen to account for differences in total intracranial volume. Further details of the MRI analysis procedure are described in the Supplementary material.

We compared grey matter volume between PPA subtypes and controls by means of vowelwise one-way ANOVA with grey matter volume as dependent variable and four subgroups (NFV, SV, LV and controls) as between-subjects factor and age as a covariate of no interest.

## Results

### Behavioural data: psychoacoustic test scores

**Figure 3:**
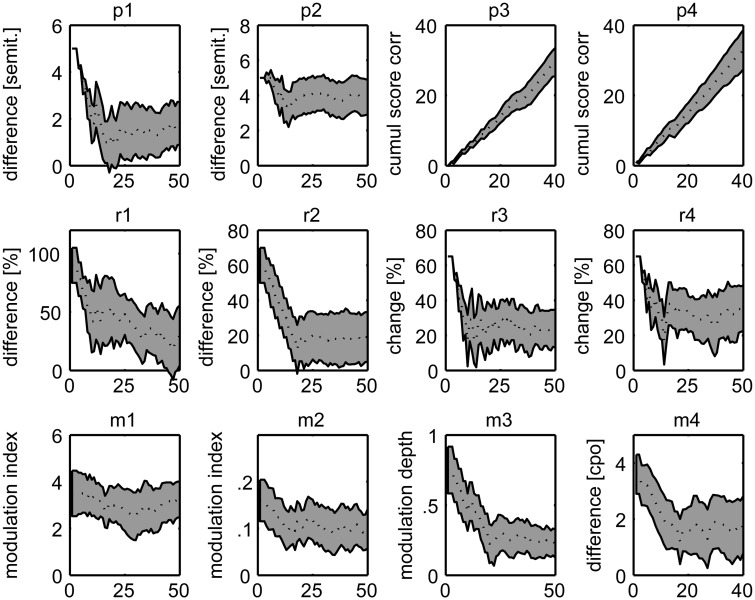
**Illustration of overall reliability for the 12 psychoacoustic experimental measures.** Depicted is the course of all patients’ individual responses as a function of trial number. The dotted line and grey shaded area depict the median response course ± the mean absolute difference from that for all patients. For the 10 adaptively controlled tasks (all except the pitch local and global sequence tasks), the graphs demonstrate the reliable course with a general decrease of respective difference between reference and target (ordinate: in pitch, or time, or modulation), and the reaching of a plateau, typically from trial 25 onward. For the two non-adaptive tasks of local and global change in pitch detection (*top right*), the graphs demonstrate the steady increase of the score correct from first to last trial.

All patients were able to perform the psychoacoustic tasks and produce informative results. Figure 3 provides overlay plots of the patients’ individual staircase tracks for the adaptive tasks, and of the cumulative score correct for the fixed-difficulty level tasks p3 and p4. Thresholds could be reliably measured in the adaptive tasks, as demonstrated by the graphs levelling off gradually and reaching a plateau, typically from trial 25 onward. In the two pitch sequence tasks (p3, p4), the constant, steady increase over the course of the tasks (40 trials) evidences the reliable measurement of discrimination ability also for these two tasks.

All outcome measures were normally distributed in both the controls and the patients in either their original form, or else after log transformation (for p1, r1, r2, m2 and m3), except one minor deviation in the patient group for r2 (*P* = 0.0175).

In the healthy controls, a multiple linear regression analysis with age and Coloured Progressive Matrices scores as independent variables and psychoacoustic test scores as outcome variable, indicated a significant correlation between Coloured Progressive Matrices scores and scores on discrimination of direction of change in pitch (p2) (Pearson r = 0.45, *P* = 0.019) but not with any of the other tasks (*P* > 0.17). There was no correlation with age.

### Experimental auditory performance: group-based analysis

**Figure 4:**
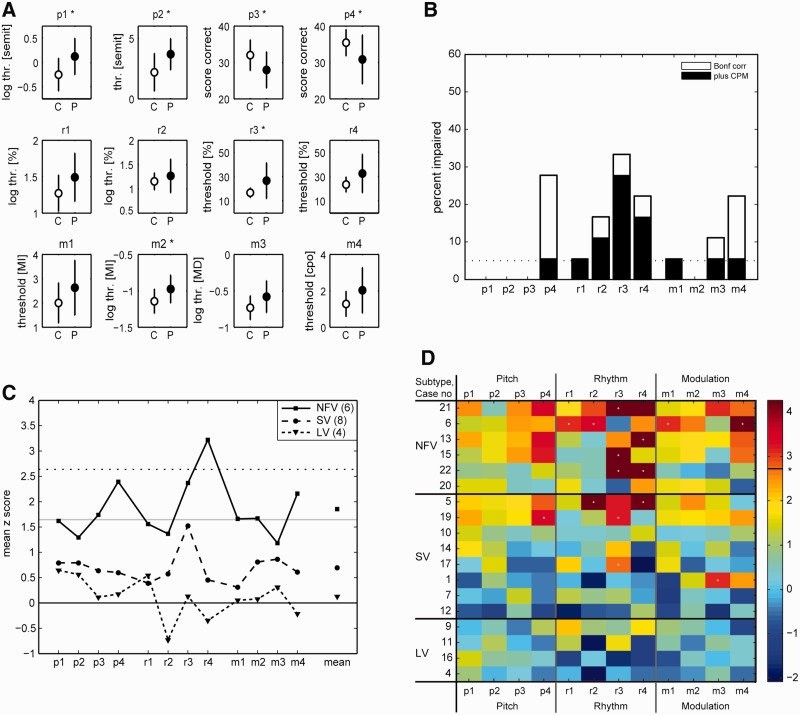
**Patients’ psychoacoustic data in comparison to controls.** (**A**) Group comparison of psychoacoustic scores between the PPA group and controls. Asterisks denote a significant difference (*P* = 0.05, Bonferroni-corrected). (**B**) Percentage of all PPA patients (*n* = 18) who exhibited a significant impairment, plotted for each of the 12 tests. White boxes indicate percentage of deficits at the individual deficits (Crawford *t*-test, Bonferroni-corrected); black boxes indicate the percentage that remains significant after regressing out the effect of Coloured Progressive Matrices (CPM) scores as a measure of fluid intelligence. (**C**) Mean Z scores across patients within the three clinical subtype groups. Mean Z scores are plotted for each task, and in addition the overall mean across tasks (*far right*). Grey line corresponds to the uncorrected *P* of 0.05, the dotted line to the Bonferroni-corrected *P* of 0.05. (**D**) All 18 individual patients’ performance in comparison to controls, in the order of severity (measured by the individual mean Z score across tasks) within each clinical subtype. The colour scale reflects the Crawford-based Z scores derived from the modified *t*-test (line and asterisk indicate Bonferroni-corrected *P* of 0.05). The deficits that remain significant (Bonferroni-corrected) after adjustment for Coloured Progressive Matrices scores are marked with a light-grey dot at the centre.

Performance in the PPA group was significantly poorer than in the control group for all pitch tasks (p1–4), the discrimination of strongly metrical patterns (r3), and FM detection at 40 Hz (m2) (Fig. 4A). When each of the subgroups was compared to the control group, discrimination of weakly metrical sequences (r4) was significantly impaired in NFV (corrected *P *< 0.05) (Fig. 4C).

We directly compared the psychoacoustic measures between the three clinical subtype groups (SV, NFV, LV) by means of the Kruskal-Wallis one-way ANOVA by ranks. This revealed a significant between-subtype difference for the detection of local [p3: χ^2^(2) = 6.03, *P* = 0.049] and global changes in pitch sequences [p4: χ^2^(2) = 6.01, *P* = 0.049] and for the discrimination of weakly metrical sequences [r4: χ^2^(2) = 9.36, *P* = 0.0093]. *Post hoc* comparisons revealed a significant difference between NFV and LV and between NFV and SV for discrimination of weakly metrical sequences (r4), with scores in NFV being more affected than in the two other subtypes (Tukey-Kramer *P* < 0.05).

### Experimental auditory performance: individual auditory profiles

Based on the psychoacoustic test scores, the SVM classifier was able to correctly assign individual cases to the NFV versus LV class in 77.6% (*P* = 0.026). This means that out of the 10 cases (NFV plus LV), 2 or 3 were misclassified per iteration. Classification between other subtypes was not possible (*P* > 0.13). The vector weights used for the classification between NFV and LV were highest for the detection of local changes in pitch sequences (p3) and for the discrimination of a weakly metrical pattern (r4). The feature weights for all 12 psychoacoustic measures are provided in Supplementary Table 2.

Significant impairments after correction for the number of tasks performed (Z > 2.64) were observed at the individual level mainly for global change in pitch detection (three NFV, two SV cases), isochrony deviation (two NFV, one SV), strongly metrical pattern discrimination (three NFV, three SV), weakly metrical pattern discrimination (three NFV, one SV) and dynamic modulation discrimination (four NFV) (Fig. 4B and D). After also adjusting for Coloured Progressive Matrices scores (Crawford and Garthwaite, 2006), significant individual impairments remained mostly for the isochrony deviation task (r2) and the metrical pattern discrimination tasks (r3–4) (Fig. 4D, indicated by light-grey dots): Three of six NFV cases were significantly impaired on weakly metrical pattern discrimination (r4) and three on strongly metrical pattern discrimination (r3). Two SV cases were significantly impaired on strongly metrical pattern discrimination (r3) (Fig. 4D).

### Relationship with neuropsychological test scores

Factor analysis of the neuropsychological test battery yielded two factors, together explaining 75.7% of the variance (Table 2). The first factor clustered naming, comprehension, object identification and semantic test scores. The second factor clustered PALPA pseudoword and word repetition scores, AAT total repetition scores and Coloured Progressive Matrices (Table 2). The outcome of this factor analysis was in line with that obtained in previous PPA studies using a similar neuropsychological protocol in independent patient groups (Vandenbulcke *et al.*, 2005; Nelissen *et al.*, 2010). Individual scores on Factor 2 (the ‘repetition factor’) correlated with scores on the detection of a local change in pitch sequence (p3: Pearson r = −0.66, corrected *P *= 0.024). There were trends in the same direction for detection of a global change in pitch sequence (p4, uncorrected *P *= 0.008), metrical pattern discrimination (r3–4, uncorrected *P *< 0.01) and three of the timbre processing tasks (m2–4, uncorrected *P* < 0.02). We did not find significant correlations between any of the psychoacoustic measures and individual scores on Factor 1.

**Table 2:** Factor analysis

Factor	Factor 1	Factor 2
Eigenvalue	7.938	2.656
Variance explained	56.701	18.969
AAT written word-picture matching	**0.903**	0.187
Boston Naming Test	**0.921**	0.100
AAT auditory word-picture matching	**0.889**	0.132
PPT	**0.873**	0.163
PALPA auditory word-picture matching	**0.859**	0.051
Object Decision A hard	**0.794**	0.156
PALPA associative-semantic task	**0.739**	0.371
Object Decision B Easy	**0.729**	0.288
AAT written sentence-picture matching	0.565	0.568
AAT auditory sentence-picture matching	0.592	0.394
PALPA pseudoword repetition	0.134	**0.912**
AAT repetition	0.047	**0.863**
CPM	0.174	**0.799**
PALPA word repetition	0.247	**0.774**

The neuropsychological test score loadings onto two factors, a naming and comprehension factor (Factor 1) and a repetition factor (Factor 2) are listed in Column 1 and 2, respectively. Loadings >0.70 are indicated in bold.

AAT = Aachen aphasia test; CPM = Coloured Progressive Matrices; PPT = Pyramids and Palm Trees test.

### Relationship with symptom duration, hearing loss and auditory short-term memory

Symptom duration did not correlate with any of the psychoacoustic test scores (*P *> 0.12) in this sample. No correlations were found between scores on any of the psychoacoustic tests and the patients’ hearing levels derived from the audiogram (Supplementary Table 1) (*P* > 0.13). Digit span forward scores correlated significantly with the scores for metrical pattern discrimination with a strongly metrical beat (r3, rho= −0.70, corrected *P* = 0.031) (Supplementary Fig. 2).

## Discussion

Using non-linguistic stimuli we performed a systematic investigation of auditory processing in the pitch, rhythm and timbre domain in PPA at a level of processing that does not depend on any association with long-term memory, emotion or meaning. In a substantial number of PPA patients, in particular those suffering from NFV, processing of short sequences (four to seven tones) of non-linguistic stimuli was found to be significantly impaired (Fig. 4C and D). This is remarkable as the clinical phenotype in NFV is dominated by speech output problems and our tests assessed the cerebral processing of auditory input.

### Differences between PPA subtypes

The non-fluent variant PPA patients (Fig. 4B and D) showed the most pronounced impairment in processing rhythm of tone sequences among the three subtypes. Within the PPA NFV subgroup, considerable phenotypical heterogeneity existed. In some NFV cases (such as Case 6), speech apraxia dominated the clinical picture (Josephs *et al.*, 2013). Other NFV patients showed prominent agrammatism in addition to speech apraxia, and yet others had word comprehension deficits in addition to the speech apraxia and agrammatism (Table 1) (Mesulam *et al.*, 2012, 2014). Regardless of this clinical variability, the psychoacoustic deficits were found across the spectrum of NFV. The psychoacoustic profile of the cases that could be classified as ‘mixed variant’ did not differ from the other NFV cases (Fig. 4D).

Only relatively recently was the LV subtype formally set apart from the NFV subtype (Gorno-Tempini *et al.*, 2004, 2011): the most characteristic deficit in LV is abnormal repetition of complex sentences, while the most characteristic deficit in NFV is motor speech apraxia and/or agrammatism. Our sound perception data allowed reliable discrimination of NFV from LV cases even though these contained no language stimuli and were purely based on perceptual judgement of pitch and rhythm in tone sequences. The language deficit in LV has been attributed to a deficit in the phonological loop implicated in models of working memory (Gorno-Tempini *et al.*, 2008; Foxe *et al.*, 2013). The relative preservation of processing of tone sequences in LV underscores the distinction between phonological working memory and the type of auditory working memory required to process non-verbal sounds and sequences of those. In this respect it is worth noting that in a previous study of intonation discrimination (Rohrer *et al.*, 2012), LV performed worse than NFV. Discrimination of pitch, duration and intensity of syllable pairs and contour discrimination of four-syllable sequences was impaired in both subtypes (Rohrer *et al.*, 2012) leading to linguistic and emotional receptive dysprosody (Rohrer *et al.*, 2012) and impaired judgements of accents (Fletcher *et al.*, 2013). These tasks all used linguistic stimuli (syllables) and this could potentially explain the difference in outcome with our study.

Previous studies have probed some of the same processes we studied but mainly in isolation, mostly with the goal of understanding higher-level processing deficits (Warren *et al.*, 2005; Goll *et al.*, 2010, 2011). Discrimination of spectral shapes is impaired in NFV (Warren *et al.*, 2005; Goll *et al.*, 2010, 2011). In our study, the most discriminative feature between NFV versus the other subtypes came from the pitch and rhythm tasks using sequences of 4–7 tones, a type of task that had not been reported on before in PPA. To the best of our knowledge, our study is the first to reveal abnormalities in pitch and rhythm processing in PPA using strictly non-linguistic stimuli.

Across the entire PPA group, the factor analysis did not indicate any relationship between the psychoacoustic test scores and anomia or tests of semantic processing. We interpret this as evidence that the processes underlying the auditory deficits are more related to a dorsal processing route, which mediates repetition of pseudowords among other tasks, than to a ventral processing route that mediates intelligibility of speech (Ueno *et al.*, 2011). This conforms with the primary aim of the study, which was to study auditory processing at a level independent of higher-level associations. At an associative level of auditory processing, the semantic variant of PPA has previously been associated with deficits in the recognition of environmental sounds (Bozeat *et al.*, 2000), famous tunes (Hsieh *et al.*, 2011), and emotions in music (Hsieh *et al.*, 2012). In our study a subset of SV cases had significantly abnormal scores on the psychoacoustic tests for pitch, rhythm and timbre depending on the individual case. Given that the stimuli used here did not convey any meaning or emotion, it cannot be said that psychoacoustic processing of non-linguistic, non-musical stimuli was entirely preserved in SV.

### Perceptual processing of pitch and rhythm in short tone sequences

The most pronounced deficits within the pitch and time domain were seen when sequences were used, and mostly so in NFV, in particular in the time domain. These striking deficits are remarkable in that the clinical picture in NFV is characterized by speech output problems and these subjects showed the most prominent perceptual deficits. A precedent for deficits in auditory analysis associated with deficits in vocal output is provided by the cortical disorder of tone deafness (Griffiths, 2008). This is recognized as an output disorder (for singing) but has been characterized as a disorder of musical analysis that can be defined by abnormal pitch-sequence perception associated with cortical pathology within a temporofrontal network for pitch working memory (Griffiths, 2008).

Among the diagnostic characteristics of speech apraxia in NFV, are the abnormal lengthening of vowels and of inter-segmental intervals (with segments referring to syllables or words) as well as the unevenness in pitch (Josephs *et al.*, 2012; Ballard *et al.*, 2014). We see a striking parity between these prominent clinical speech output characteristics and the pitch, duration and timing judgement errors during the perceptual tasks we applied. Because of this apparent similarity, we favour a unifying explanation for both the speech production problem and the acoustic perceptual abnormalities. The clinically prominent motor speech problems and the subclinical auditory deficit for timing and pitch could be related via a common ‘temporal scaffolding’ of processing of acoustic structure over time, used for auditory input up to speech output (Grube *et al.*, 2013). In a broader theoretical sense, auditory perceptual processing is considered fundamental to normal speech production in several models, such as the sensory theory of speech production (Giraud and Poeppel, 2012).

The sequences did not contain linguistic stimuli and therefore our tasks relied on working memory for auditory objects rather than phonological working memory. A verbal short-term memory deficit, as measured by means of digit span forward, is present in NFV, and principally so in cases with agrammatism with or without apraxia of speech (Rohrer *et al.*, 2010). The sequence processing deficit in our study could be caused by an auditory working memory deficit distinct from phonological working memory, an observation that has not been reported before to our knowledge. We, however, consider it unlikely that an auditory working memory problem could explain the motor speech deficits in NFV but it could play a role in agrammatism (Grossman and Ash, 2004; Rohrer *et al.*, 2010). The motor speech deficit may co-occur with the non-verbal auditory working memory deficit because both rely on nearby anatomical regions. Auditory working memory relies on inferior frontal gyrus and sulcus and premotor cortex (Smith and Jonides, 1999), regions of predilection in NFV.

### Potential study limitations

PPA is relatively rare and patients can only reliably fulfill demanding psychophysical tasks of this kind when they are in a fairly early stage. Statistical comparisons between subgroups or negative findings within subgroups (such as LV) must be interpreted with caution given the relatively low sample size per subgroup. Potential differences in stage or severity of the disease may also play a role when comparing between subtypes.

Inclusion criteria were, among others, relatively preserved hearing levels (Supplementary Table 1) as well as proof of proper understanding and execution of the tasks during practice trials. The psychoacoustic tasks have been designed to avoid as much as possible interference from language production or comprehension deficits. Reliable results were obtained, as illustrated by the adaptive threshold tracking staircase plots and the cumulative correct scores for the tasks with fixed-difficulty levels (Fig. 3). Significant deficits remained after adjustment for general cognitive capacity (executive function or fluid intelligence), as measured with Raven’s Coloured Progressive Matrices.

Our battery did not include a formal speech apraxia assessment. The repetition tests require subjects to repeat phonemes, consonant-vowel combinations, words, pseudowords and sentences. The pronunciation is scored in fine detail, so that patients with motor speech deficits will be impaired to varying degrees on these tests (Sajjadi *et al.*, 2012; Leyton *et al.*, 2014) (Table 1).

Finally, it is inherent to this type of correlational study that we cannot establish a causal link between e.g. the perceptual deficit and the motor speech deficit as this would logically require an effective intervention.

## Conclusion

While PPA NFV is clinically characterized by a motor speech output deficit, our findings clearly indicate a concomitant problem with perceptual processing of timing of tone sequences. We therefore argue that accurate sequence processing of timing is necessary for both motor speech and perceptual judgements of tone sequences. Both the motor and the perceptual parts may rely on a common ‘scaffolding’ of processing of acoustic structure over time, used for auditory input up to speech output (Grube *et al.*, 2013).

## Supplementary Material

Supplementary Data

## Abbreviations

LV =: logopenic variant
NFV =: non-fluent/agrammatic variant
PALPA =: Psycholinguistic Assessment of Language Processing in Aphasia
PPA =: primary progressive aphasia
SV =: semantic variant
