# Supplementary material for: Core auditory processing deficits in primary progressive aphasia
Source: Brain. 2016 Apr 9;139(6):1817–29. doi: 10.1093/brain/aww067 (PMC4892752; doi:10.1093/brain/aww067)

Supplementary Materials Methods

Neuropsychological protocol

Confrontation naming was assessed by means of the Boston Naming Test (BNT) (Kaplan et al., 1983, Marien et al., 1998). We assessed single word comprehension and repetition using tests selected from the Psycholinguistic Assessment of Language Processing in Aphasia (PALPA) (Kay et al., 1992, Bastiaanse et al., 1995) and the Akense Afasie test (Graets et al., 1992) (Table 1). In the word-picture matching task (PALPA item 45), a concrete noun is presented auditorily together with a target picture and 4 distractors. Two distractors are semantically related to the target, a third is perceptually similar and the fourth picture is unrelated. Subjects have to point to the target. In the associative-semantic task (PALPA 49), a written noun is presented together with 4 other written choice noun stimuli (a target noun, a noun that is semantically related to the target, and two unrelated nouns) and subjects have to underline or circle the noun that matches the sample stimulus most closely in meaning, for a total of 15 word series with high imageability and 15 with low imageability. In the auditory and written comprehension subtests of the AAT, 60 words and 60 sentences are presented per modality. One target and three distractor picture stimuli are presented simultaneously and subjects have to indicate the picture that corresponds to the word or the sentence, respectively. In the PALPA word repetition task (PALPA 9), the examiner who is facing the subject, pronounces 80 nouns and 80 pseudowords which the subject has to repeat. In the AAT repetition task, the examiner pronounces 10 phonemes (e.g. /a:/), 10 monosyllabic words (e.g. 'oost'), 10 foreign words (e.g. 'kitsch'), 10 composed words (e.g. 'voorlichtingsgesprek') and 10 sentences of increasing length (e.g. from 'de hond blaft' to 'hij haalde zijn moeder met de nieuwe auto van het station af'), which the subject has to repeat. Repetition on the AAT test is rated per item from 0 (wrong), 1 (minor mistake), 2 (minimal mistake) to 3 (entirely correct). Subjects also received the 1-minute animal verbal fluency test, the picture version of the Pyramids and Palm

Trees test, the Raven's Colored Progressive Matrices (CPM) as a nonverbal measure of fluid intelligence (executive domain), and the Birmingham Object Recognition Battery Object Decision test, as a measure of object identification.

Experimental tests of pitch, rhythm and timbre

The psychoacoustic battery is closely similar to that used by Grube et al. (2012, 2014). Graphical, schematic illustrations of tasks and stimuli are provided in Figure 1. Sound was generated by means of a Roland UA-4FX audio/MIDI interface with Sennheiser HD linear 250-II/280 headphones. Sound intensity was adjusted individually from a fixed value to a comfortable level where needed, within a range of 65 to 80 dB SPL.

Adaptive tracking procedure and threshold estimation

The tasks were implemented in Matlab (version 7.2.0 Mathworks, 2006). Depending on the task, one trial comprised two or three stimulus intervals, with each interval consisting of one sound, a pair of sounds, or a sound sequence. The tasks followed either an AB, an AXB, or an XAB paradigm. A and B denote two intervals containing the target and a reference stimulus, respectively, in random order and with equal probability. The AB tasks can either be same-different tasks or target identification tasks. X corresponds to an additional interval containing another reference-like stimulus in some of the discrimination tasks. Inter-stimulus-interval and inter-trial-interval (from a subject's response) varied between 500 and 2000 ms per task.

The majority of tasks (two pitch, all rhythm, all timbre) were based on a two-alternative forced-choice adaptive paradigm following a 2-down, 1-up algorithm (difficulty increasing after 2 consecutive correct responses and decreasing after every incorrect one). A larger step size was used up to the fourth reversal and after that a smaller one. The outcome measure was the threshold, calculated as the mean over the last six reversals measured with the small step size, estimating the 70.9%-correct point of the psychometric function (Levitt, 1971). Two pitch

tasks used fixed difficulty levels (Foxton et al. 2003), with the score correct as the outcome measure.

Depending on preference and ability, subjects responded by pressing keys on the keyboard, verbally, or by pointing to the corresponding interval on a graphical scheme reflecting the trial structure (a piece of paper placed in front of them showing two or three circles numbered 1, 2 and 3). Each subject responded using the same response modality for all tasks.

Prior to each task, we explained to the subject which domain (pitch etc.) was being probed. The instructions were repeated to the subject as needed, until they confirmed to have fully understood the task. We presented the instructions verbally, and also in a graphical way and by simple examples (Supplementary Figure 1). Next, subjects received a number of practice trials. The difficulty level of the practice trials was the same for all subjects at the start for each task. If an incorrect response was given on a practice trial, the subject received feedback on the nature of the error. Practice trials and instructions were repeated until 5 consecutive correct responses were obtained from the subject. If needed, the intitial difference (and step sizes) were increased individually in patients in order to measure their perceptual thresholds reliably.

The difficulty level at the start of the test was the same as in the practice trials. If the subject indicated during the test that they had forgotten the instructions, the task was aborted, the instructions repeated, practice trials run again and the test re-started.

Pitch

The basic *change in pitch detection* task (p1; 50 trials; AB) (Fig 1A) required pairs of pure tones to be discriminated based on their frequency. On each trial, two pairs of tones (duration, 250 ms each) were presented. The target pair to be identified by the subject contained a change in frequency (up- or downward), the reference did not. The centre frequencies for the two pairs were roved independently in the range of 500 Hz ±3 semitones in a pseudo-randomized, fixed

orders. The magnitude of the change in frequency within the target pair had a default starting value of 5 semitones, adaptively adjusted in steps of 0.8 and 0.2.

The *pitch change direction discrimination* task (p2; 50 trials; AB) (Fig 1A) required two pairs of pure tones to be judged as 'same' or 'different' based on the direction of changes in frequency within the pairs. The frequency within a pair could either go 'up' or 'down', and the two pairs could thus have the 'same' or 'different' directions. The combination of directions was pseudo-randomized in a balanced, fixed order across trials, with equal numbers of 'same' and 'different' pairs going 'up' and 'down'. The centre frequencies for the two pairs were in the range of 500 Hz +/- 3 semitones, roved between trials and pairs. The change in frequency within pairs had a default starting value of 5 semitones, adaptively adjusted in steps of 0.8 and 0.2.

The *local (p3) and global (p4) change in pitch detection* tasks (40 trials; AB) (Fig 1A) used pitch sequences of 4 tones (duration, 250 ms), and required the subject to indicate whether two sequences were 'the same or different' (adapted from Foxton et al. (2003)). In the *local* task, in the “different” pairs, there was one change in frequency in the third or fourth note in the second sequence compared to the first, but the patterns of ‘ups and downs’ remained preserved. In the *global* task in contrast, the change in frequency also caused a change in pitch contour, i.e. the pattern of 'ups and downs'. Either task included 20 'same' and 20 'different' trials, based on 20 reference sequences, each occurring once in a 'same' and in a 'different' trial, in a pseudo-randomized fixed order.

Rhythm and timing

All four rhythm tasks (Fig. 1B) used 500-Hz, 100-ms tones.

The *single time-interval* duration discrimination task (r1; 50 trials; AB) (Fig. 1B; Grube et al., 2010) required subjects to indicate which of two tone pairs comprised the 'longer gap'. The reference pair had an inter-onset-interval of 300 to 600 ms (roved in 60 ms steps, in pseudo-

randomized, fixed order). The initial default difference in the target being longer was by 90% of the reference inter-onset-interval, and adaptively adjusted in steps of 12% and 6%.

In the *isochrony deviation detection* task (r2; 50 trials; AB) (Fig. 1B; Grube et al., 2012), subjects were required to indicate which of two otherwise isochronous 5-tone sequences contained a lengthening or ‘extra gap’. Both sequences had an isochronous inter-onset interval ranging from 300 to 600 ms (roved in 60 ms steps), and the target one lengthened interval after the 3rd tone. The initial default value of the lengthening was 60% of the inter-onset-interval, adaptively adjusted in steps of 6% and 2%.

In the *metrical pattern discrimination* tasks (r3, r4; 50 trials; XAB) (Fig. 1B, Grube and Griffiths, 2009), subjects were required to decide which of three 7-tone rhythms sounded 'different' or 'wrong'. The reference sequence had a strongly (r3) or a weakly (r4) metrical beat of 4 evoked by the temporal spacing of the tones over 16 time units of 180-220ms each (roved between stimuli). In the strongly metrical sequence, accented tones occurred every 4 unit, in the weakly metrical sequence, two of those were silent (for more details see Grube and Griffiths (2009)). The default initial distortion in pattern (a change in the long compared to the short intervals) was 65%, adaptively adjusted in steps of 12% and 6%.

Modulation

In the two *FM detection* tasks (m1, m2; 50 trials each; AB) (Grube et al., 2012), subjects were required to identify a target tone modulated in frequency at a rate of 2 Hz (m1; sounding 'ringing or wobbly') or 40 Hz (m2; sounding 'rough'), against a 'flat-sounding' unmodulated 500 Hz reference. Sound duration was 1000 ms. The initial magnitude of modulation, expressed by the modulation index (MI: ratio of maximum frequency deviation to modulation frequency) was 3.5 for the 2 Hz FM (equalling ± 7 Hz maximum deviation from the carrier frequency of 500 Hz), adapted in steps of 0.39 and 0.13. For the 40 Hz FM, the default initial MI was 0.16

(equalling ± 6.4 Hz maximum frequency deviation for the carrier), adapted in steps of 0.13 and 0.06.

The *DM detection* task (m3; 50 trials; AB) (Grube et al., 2012) required the discrimination of a dynamically modulated ('alien or laser-like') target sound against an unmodulated reference (duration, 1000 ms). Sounds comprised 100 components per octave (logarithmic spacing) over 4 octaves (250-4000 Hz). Components' amplitudes were sinusoidally modulated in frequency (spectral) and time (temporal) with a rate of 1.5 cycles per octave (cpo) and 8 cycles per second (Hz), respectively. These rates also occur in speech (Chi et al. 1999), but the sounds have no resemblance to any natural speech sounds. All stimuli had an upward drift in spectral peaks over time. Default initial modulation depth was 0.75, adaptively adjusted in steps of 0.075 and 0.025.

The *DM discrimination* task (m4; 50 trials; AXB) (Grube et al., 2012) required the discrimination of a target sound with a different spectro-temporal modulation than two reference sounds. Reference stimuli had a spectral rate of 1.5 cpo and a temporal rate of 8Hz, the target a higer spectral rate (modulation depth, 0.75; intensity, roved by). The default initial difference in spectral modulation rate was 3.6 cpo, adapted in steps of 0.4 and 0.2.

Machine-learning classifier

We examined how accurately a machine learning classifier could assign an individual case to one of the 3 clinical subtype groups (three classes: NFV, SV and LV) based on the set of 12 psychoacoustic test scores, regardless of any other behavioral or imaging information, and which of the psychoacoustic tests were most discriminative in that respect. We used a linear support vector machine (SVM) approach (C= Inf, alpha = 0) as implemented in Spider (version 1.71 (http://people.kyb.tuebingen.mpg.de/spider/, Weston J., Elisseef A, BakIr G., and Sinz F., Max Planck Institute for Biological Cybernetics, Tübingen, Germany, running under Matlab

version 2011b) for pairwise classification into every possible pair of subtypes (NFV-LV, NFV-SV, LV-SV). Half of the data for every subtype was used for training the classifier, while the other half was used as a test set to determine the performance of the classifier (twofold cross-validation). Multiple repeats (n = 1400) were performed in a way so that each possible combination of training and test data was used. Next, we calculated if the performance of the classifier was better than expected by chance, using random permutation labelling. For this purpose, PPA subtype labels were randomly assigned and the classification procedure was run 1,000 times as described above. Finally, we determined the feature weights for each psychoacoustic test. The feature weight of a psychoacoustic test is the component of the normal vector of the hyperplane along that test's dimension. Feature weights indicate how much a particular test contributes to the classification.

Factor analysis of the neuropsychological test scores

We further tested for a relationship between the psychoacoustic test scores and conventional neuropsychological measures. Given the relatively large number of the conventional neuropsychological tests administered and the potential correlations between scores on these conventional tests, we first conducted a factor analysis (SPSS Statistics 22, IBM) on the full neuropsychological dataset to reduce the number of correlations to be performed with the psychoacoustic scores. The dataset included the conventional neuropsychological test scores (Table 1) from all PPA patients, plus the 28 age-matched controls who had undergone the same neuropsychological battery. The factor analysis procedure was identical to that used in previous studies (Vandenbulcke et al., 2005, Molenberghs et al., 2009, Nelissen et al., 2010). Factors were extracted consecutively and orthogonalized to each other. Only factors with an eigenvalue > 1 were retained (Kaiser criterion). A variance maximizing (varimax) rotation of the original variable space was used to obtain an interpretable pattern of factor loadings while preserving factor orthogonality. Factor

scores for every subject were derived from these loadings and indicate how well a subject performed on the neuropsychological tests subsumed under a given factor. Individual factor scores were calculated per factor. Normality of the factor scores was tested using the Lilliefors version of the Kolmogorov-Smirnov Test for Composite Normality. The individual factor scores of the PPA cases were correlated with their scores on the psychoacoustic tasks using the Pearson correlation method throughout. The significance threshold was set at one-tailed $P < 0.05$, Bonferroni-corrected for multiple comparison by the number of tests (n = 12 times the number of factors).

Volumetric MRI

All patients, except case 10 (claustrophobia), and 86 healthy controls received a high resolution T1-weighted structural MRI on a 3T Philips Intera system equipped with an 8-channel receive-only head coil (Philips SENSitivity Encoding head coil), using a 3D turbo field echo sequence (coronal inversion recovery prepared 3D gradient-echo images, inversion time (TI) 900 ms, shot interval = 3000 ms, echo time (TE) = 4.6 ms, flip angle 8 degrees, field of view (FoV) = 250x250 mm, 182 slices, voxel size 0.98 x 0.98 x 1.2 mm^3).

MRI analysis

All procedures were carried out with Statistical Parametric Mapping 8 (SPM8, Wellcome Trust Centre for Neuroimaging, London, UK, (http://www.fil.ion.ucl.ac.uk/spm) and the Voxel-Based Morphometry 8 toolbox (VBM8, http://dbm.neuro.uni-jena.de/vbm). High-resolution T1-weighted images were corrected for bias-field inhomogeneities, registered to the Montreal Neurological Institute (MNI) space using linear (12-parameter affine) and non-linear transformations, and segmented into grey matter, white matter, and cerebrospinal fluid within the same generative model (Ashburner and Friston, 2005). An erosion/dilatation procedure with thorough cleaning removed most of the extracerebral voxels. Misclassified voxels were

manually removed and T1-weighted images were reentered into the VBM routine. The normalized grey matter partitions were weighted ('modulated') to account for non-linear volume changes resulting from the normalization process, allowing the comparison of absolute amount of tissue corrected for individual brain sizes. The voxel size of the images in MNI space was 1.5 x 1.5 x 1.5 mm^3. Modulated grey matter maps were smoothed with an 8 x 8 x 8 mm^3 full-width at half-maximum (FWHM) Gaussian kernel. All preprocessed grey matter maps passed visual inspection for overall segmentation and normalisation accuracy. Smoothed modulated grey matter maps were entered into the statistical analyses masked with an absolute threshold of 0.1.

Supplementary Tables

Case	Left ear hearing level (dB) 250 Hz	500 Hz	1000 Hz	2000 Hz	4000 Hz	Mean	Right ear hearing level (dB) 250 Hz	500 Hz	1000 Hz	2000 Hz	4000 Hz	Mean
1	15	20	25	**35**	**65**	32	10	20	25	25	**65**	29
5	**50**	**55**	**50**	**45**	**85**	57	10	15	15	15	30	17
7	-	5	1	10	**60**	19	-	10	1	10	**40**	15,25
10	10	10	5	15	20	12	15	10	15	20	**35**	19
12	15	10	1	20	**70**	23,2	15	10	5	20	**65**	23
14	10	10	10	1	15	9,2	10	10	10	1	15	9,2
17	25	30	30	30	**45**	32	20	30	30	25	30	27
19	**60**	**40**	30	**40**	**80**	50	20	10	15	1	**50**	19,2
6	5	1	5	1	10	4,4	10	15	10	5	20	12
13	5	5	10	1	5	5,2	5	1	10	1	15	6,4
15	15	10	25	**35**	**70**	31	15	5	10	**35**	**50**	23
20	**65**	**60**	**75**	**70**	**70**	68	10	30	30	**40**	**60**	34
21	5	10	15	15	**50**	19	15	10	5	20	30	16
22	10	10	15	30	**40**	21	10	15	10	15	15	13
16	5	1	5	10	25	9,2	1	5	1	5	15	5,4
4	5	5	10	5	15	8	10	5	5	10	15	9
9	10	-5	5	10	20	8	5	5	15	10	20	11
11	15	15	15	5	25	15	10	10	15	5	20	12
2	20	15	20	15	**40**	22	10	10	15	10	25	14

Supplementary Table S1: Hearing loss of patients. Frequencies which required 30 dB or more to be perceived by the patient are indicated in bold.

	NFV vs LV
**p1**	0,0053
**p2**	0,0192
**p3**	0,0612
**p4**	0,0006
**r1**	-0,0004
**r2**	0,0005
**r3**	-0,0067
**r4**	0,0898
**m1**	0,0100
**m2**	0,0028
**m3**	0,0023
**m4**	0,0104

Supplementary table S2: Feature weights of SVM used for classification between NFV versus LV. SVM was able to discriminate between NFV and LV, with highest feature weights for detection of a local change in pitch sequences (p3) and for discrimination of weakly metrical sequences (r4).

Supplementary figure 1: Graphical examples used for explaining the task instructions to the study participants

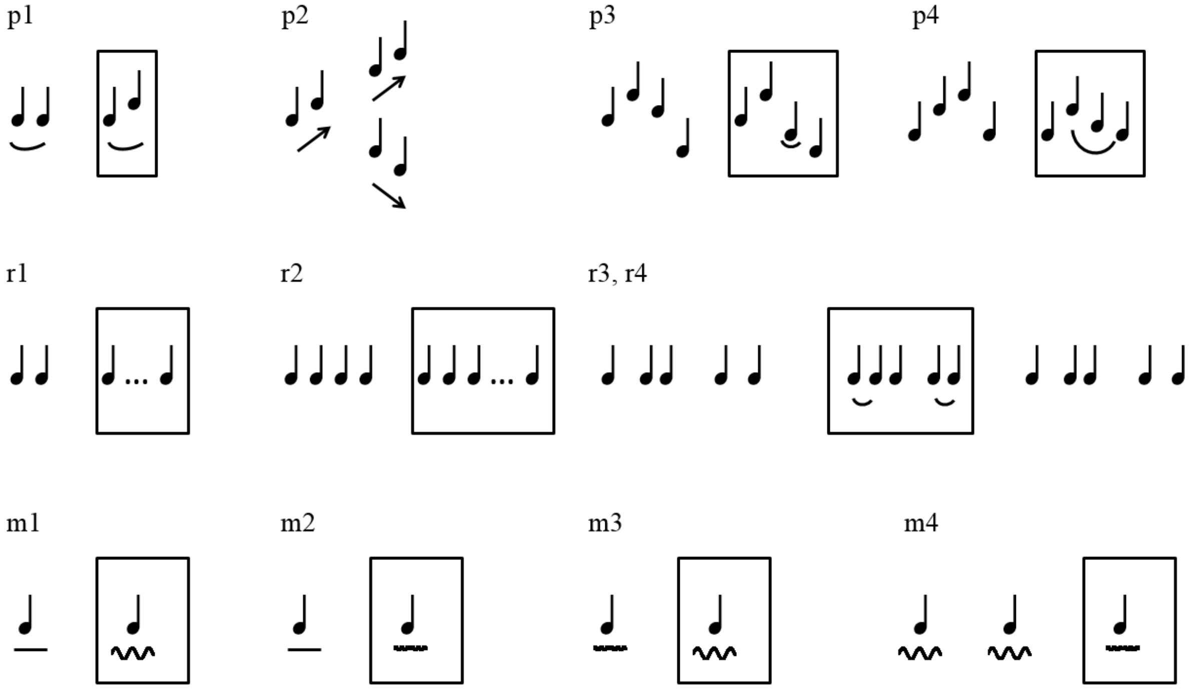

Supplementary figure 2: Regression plots for each of the psychoacoustic test scores with digit span forward as independent variable. At a corrected P level, the correlation is significant only for metrical pattern discrimination with a strongly metrical beat (r3). At an uncorrected $P < 0.05$, correlations were also present for basic change in pitch detection (p1, rho = -0.50, uncorrected $P = 0.048$), local and global change in pitch detection (p3, rho = -0.53, uncorrected $P = 0.035$; p4, rho = -0.53, uncorrected $P = 0.042$).

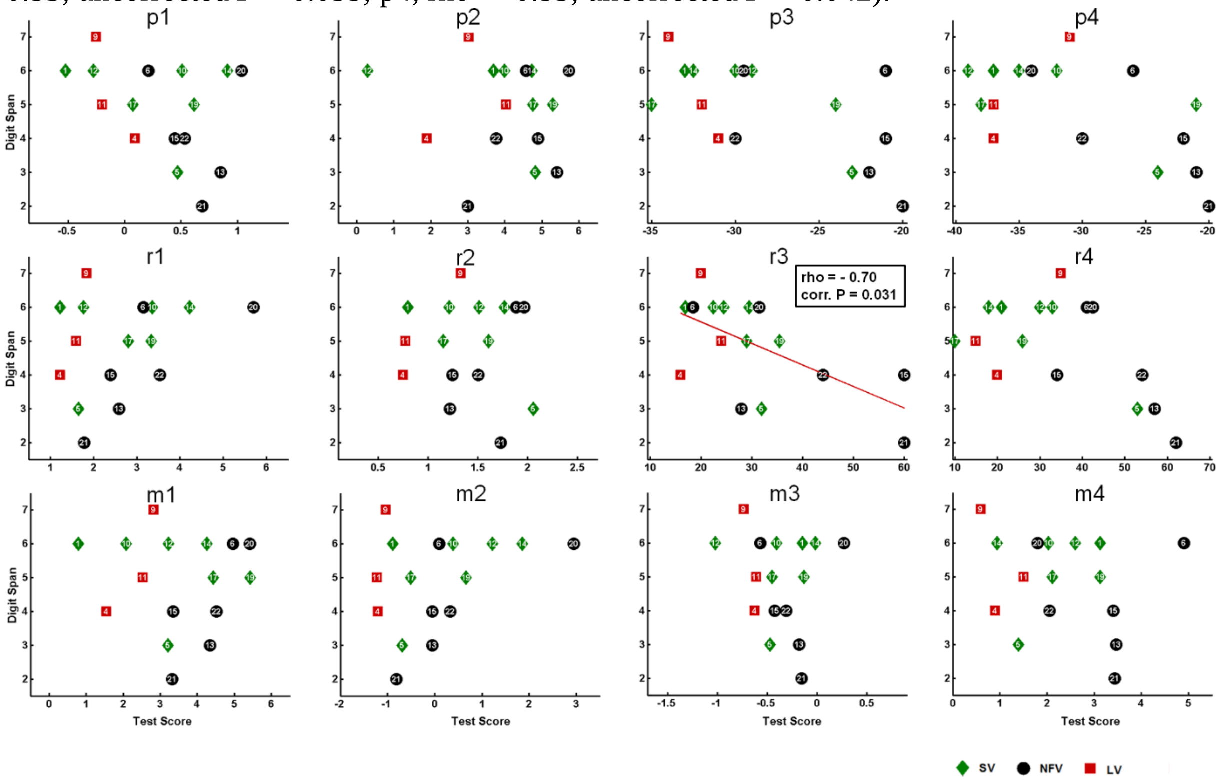

Supplement: Supplementary Data [file aww067_supplementary_data.zip › brain-2015-01247-File006.pdf]
