# Supplementary figures and images for: Core auditory processing deficits in primary progressive aphasia
Source: Brain. 2016 Apr 9;139(6):1817–29. doi: 10.1093/brain/aww067 (PMC4892752; doi:10.1093/brain/aww067)

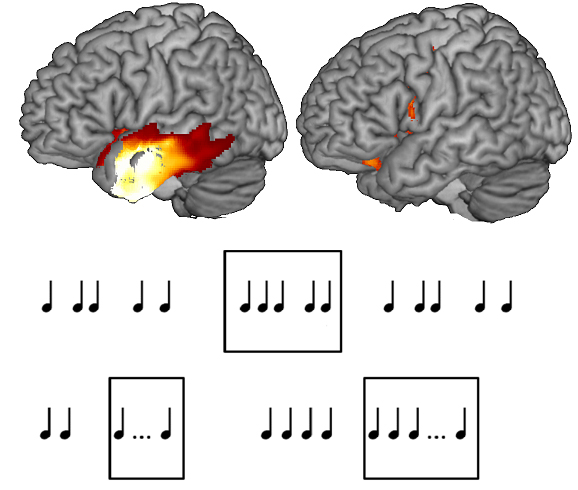

Supplement: Supplementary Data [file aww067_supplementary_data.zip › brain-2015-01247-File007.jpg]
